# Nuclear localization of tricellulin promotes the oncogenic property of pancreatic cancer

**DOI:** 10.1038/srep33582

**Published:** 2016-09-19

**Authors:** Akira Takasawa, Masaki Murata, Kumi Takasawa, Yusuke Ono, Makoto Osanai, Satoshi Tanaka, Masanori Nojima, Tsuyoshi Kono, Koichi Hirata, Takashi Kojima, Norimasa Sawada

**Affiliations:** 1 Departments of Pathology, Sapporo Medical University School of Medicine, Sapporo 608556, Japan; 2 Division of Advanced Medicine Promotion, The Advanced Clinical Research Center, The Institute of Medical Science, The University of Tokyo, Tokyo 108-8639, Japan; 3 Departments of Surgery, Sapporo Medical University School of Medicine, Sapporo 608556, Japan; 4 Department of Cell Science, Research Institute of Frontier Medicine, Sapporo Medical University School of Medicine, Sapporo 608556, Japan

## Abstract

Accumulating evidence has shown that dysregulation of tight junctions (TJs) is involved in tumor development and progression. In this study, we investigated the expression and subcellular distribution of tricellulin, which constitutes tricellular TJs, using human pancreatic adenocarcinomas. In well-differentiated pancreatic adenocarcinoma tissues, tricellulin immunostaining was prominent in the cytoplasm and the plasma membrane. In contrast, in poorly differentiated tissues, its immunostaining was predominantly observed in the nuclei and was almost absent in the plasma membrane. The distinct immunostaining of tricellulin successfully distinguished poorly differentiated adenocarcinoma from moderately and well-differentiated adenocarcinomas with high levels of sensitivity and specificity. Nuclear tricellulin expression significantly correlated with lymph node metastasis, lymphatic invasion and poor survival. In pancreatic cancer cell lines, tricellulin localization shifted from the membrane to nucleus with decreasing differentiation status. Nuclear localization of tricellulin promoted cell proliferation and invasiveness possibly in association with MAPK and PKC pathways in pancreatic cancers. Our results provide new insights into the function of tricellulin, and its nuclear localization may become a new prognostic factor for pancreatic cancers.

Pancreatic cancer shows an extremely poor prognosis with a 5-year survival rate of less than 5% due to its highly invasive and metastatic phenotypes^1^,^2^,^3^. Thus, an understanding of the regulatory mechanisms that control the aggressive behavior of this malignancy is needed.

Tight junctions (TJs) are the apicalmost components of intercellular junctional complexes in epithelial and endothelial cells. They primarily inhibit solute and water flow through the paracellular space^4^,^5^. They also separate the apical from the basolateral cell surface domains to establish cell polarity^6^. TJs are formed by not only integral membrane proteins such as claudins but also a variety of subcellular scaffolding proteins^7^,^8^,^9^. Accumulating evidence has shown that these components are signaling molecules that have functions in receiving or transmitting signals^7^,^8^,^9^. Morphological examinations of TJ proteins demonstrated that various human tumors^10^,^11^,^12^,^13^,^14^,^15^,^16^, including pancreatic cancer^17^,^18^, show aberrant expression and localization of TJ components. TJs are frequently disassembled in carcinomas with poor differentiation^19^,^20^. These findings suggested that dysregulated or disordered TJs are involved in tumor development and progression.

Tricellulin, which is encoded by the *TRIC* gene, is a transmembrane protein that predominantly localizes at tricellular TJs, where the corners of three epithelial cells meet^21^,^22^. Results of previous studies have revealed that this protein is a possible factor contributing to pancreatic neoplasia^23^,^24^, and this possibility is supported by the results of our previous study showing that tricellulin regulates epithelial TJ integrity of pancreatic duct epithelial cells via the c-Jun N-terminal kinase pathway^25^. In addition, immunohistochemical analyses have revealed that well-differentiated pancreatic adenocarcinomas highly express tricellulin in contrast to poorly differentiated carcinomas^23^. However, the potential role of tricellulin in carcinogenesis remains to be clarified.

In the present study, we examined tricellulin expression in human pancreatic cancers in association with its subcellular localization, and we evaluated possible correlations with several clinicopathological variables. We also investigated whether tricellulin expression and its subcellular localization are responsible for the aggressive behaviors of cancer cells such as proliferation and invasiveness. Our results suggest that aberrant nuclear localization of tricellulin confers immature histology and oncogenic properties of pancreatic malignancy.

## Results

### Tricellulin localization alters depending on differentiation levels in human pancreatic cancer tissues

**Figure 1:**
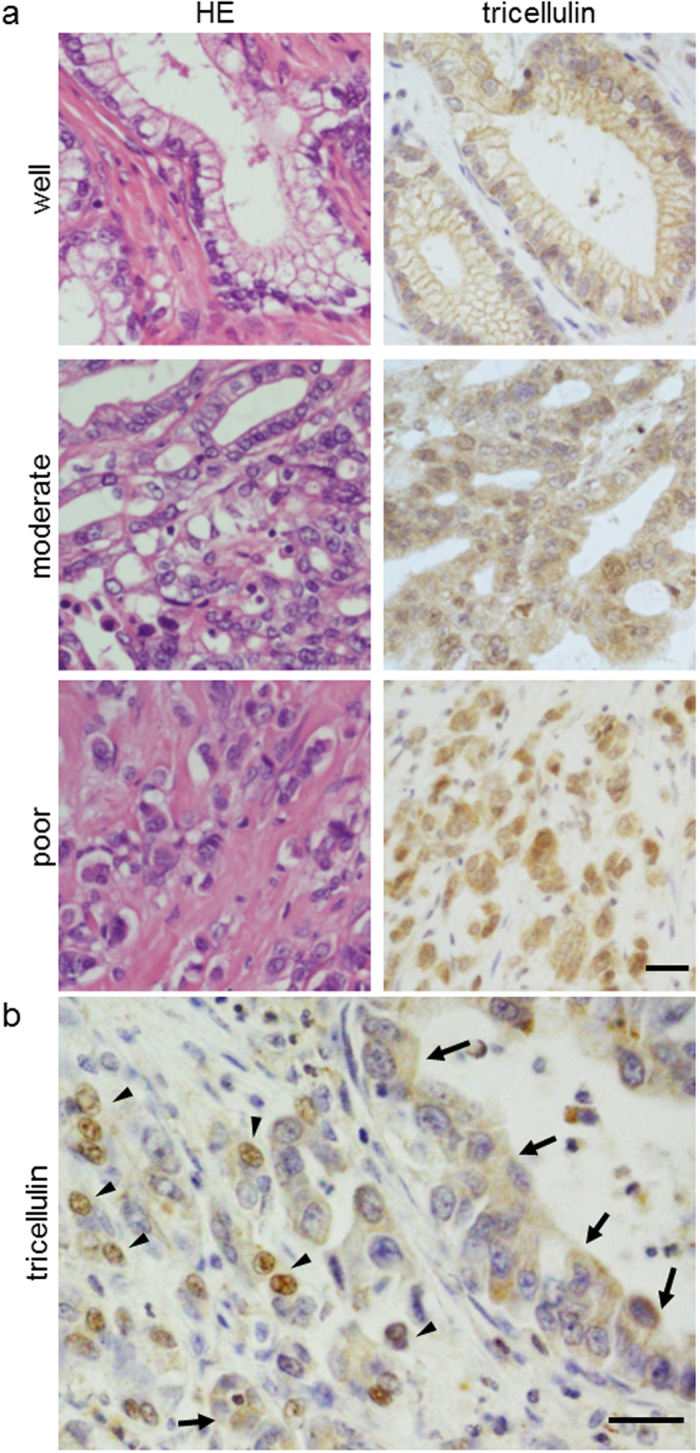
HE staining and immunohistochemical staining for tricellulin in human pancreatic adenocarcinoma tissues. (**a**) Expression of tricellulin in surgical specimens of pancreatic adenocarcinoma. Tricellulin was localized at the plasma membrane and cytoplasm in well- and moderately differentiated pancreatic adenocarcinoma, whereas it assembled in the nuclei in poorly differentiated adenocarcinoma. Bar: 30 μm. (**b**) In cases with mixed differentiation, tricellulin was localized at the cytoplasm and plasma membrane in areas with irregularly arranged lumen formation (arrows), and tricellulin was localized in the nuclei in poorly differentiated areas characterized by lack of tubule formation (arrowheads). Bar: 30 μm.

In normal pancreatic tissues, tricellulin was expressed in the intercellular boundary of pancreatic ductal cells (data not shown). In adenocarcinomas, tricellulin immunoreactivities were more prominent than in normal regions, and the subcellular distribution of tricellulin varied depending on the degree of differentiation (Fig. 1a): In well-differentiated carcinoma components, localization of tricellulin was prominent in the cytoplasm and the plasma membrane. In contrast, in poorly differentiated carcinoma components, localization of tricellulin was predominantly observed in the nuclei with various mixed patterns of cytoplasmic staining, whereas membranous staining was hardly observed. In cases with mixed differentiation, tricellulin was localized at the cytoplasm and plasma membrane in areas with irregularly arranged lumen formation, and tricellulin was localized in nuclei in poorly differentiated areas characterized by lack of tubule formation (Fig. 1b). For semi-quantitative analysis of the nuclear immunoreactivity of tricellulin, total numbers of immunopositive nuclei were counted in ten high-power microscopic fields that were randomly selected (Table 1a). The maximal score for the nucleus was significantly higher in poorly differentiated tissues.

We next performed ROC curve analysis of the nuclear immunoreactive score to determine whether it can distinguish the differentiation status of pancreatic adenocarcinomas (Supplemental Fig. 1). At a cutoff value of 21, poorly differentiated adenocarcinoma was successfully distinguished from moderately and well-differentiated adenocarcinomas with high levels of sensitivity and specificity (Table 1b), indicating that nuclear localization of tricellulin in poorly differentiated pancreatic adenocarcinoma is significantly different from that in moderately and well-differentiated adenocarcinomas.

### Nuclear localization of tricellulin is a prognostic factor of pancreatic cancers

**Figure 2:**
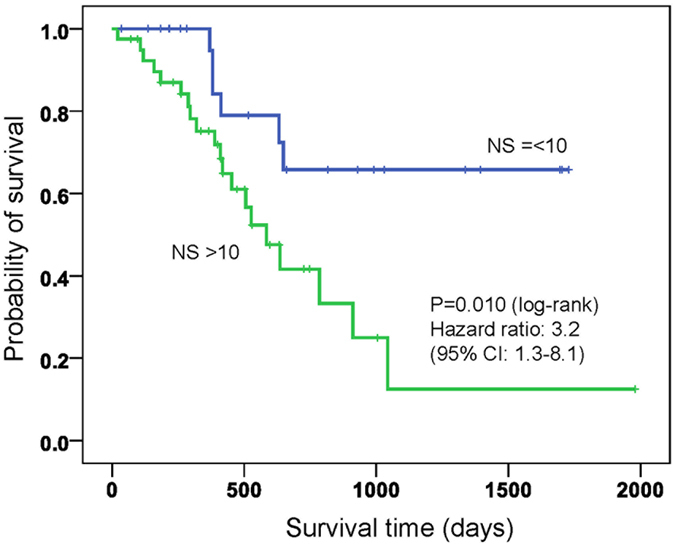
Kaplan-Meier survival curves for pancreatic cancer patients. Patients with low nuclear staining scores (NS) of tricellulin had better overall survival than did those with high nuclear staining scores of tricellulin, as defined by the log-rank test (P = 0.010). Hazard ratio is 3.2 (95% CI: 1.3–8.1).

We next examined the possible relationships between nuclear staining score of tricellulin and clinicopathological parameters. At a cutoff value of 11, nuclear tricellulin expression was significantly correlated with lymph node metastasis (pN category, p = 0.004), lymphatic invasion (p = 0.059), and vessel invasion (p = 0.057, Table 2). In contrast, nuclear localization of tricellulin was not associated with sex, age, pT status, UICC stage, vessel invasion, neural invasion or tumor location. Kaplan-Meier analysis demonstrated that patients whose nuclear staining scores were 10 or less had significantly better survival than that of patients whose scores were greater than 10 (Fig. 2, 95% CI: 1.3–8.1, log-rank P = 0.010, hazard ratio: 3.2). These findings indicated that nuclear localization of tricellulin is likely to be a prognostic factor of pancreatic cancers.

### Tricellulin accumulates in the nucleus of a poorly differentiated pancreatic cancer cell *in vitro*

To determine whether tricellulin nuclear localization is directly involved in the malignant behaviors of cancer cells, we performed *in vitro* experiments using two human pancreatic cancer cell lines.

**Figure 3:**
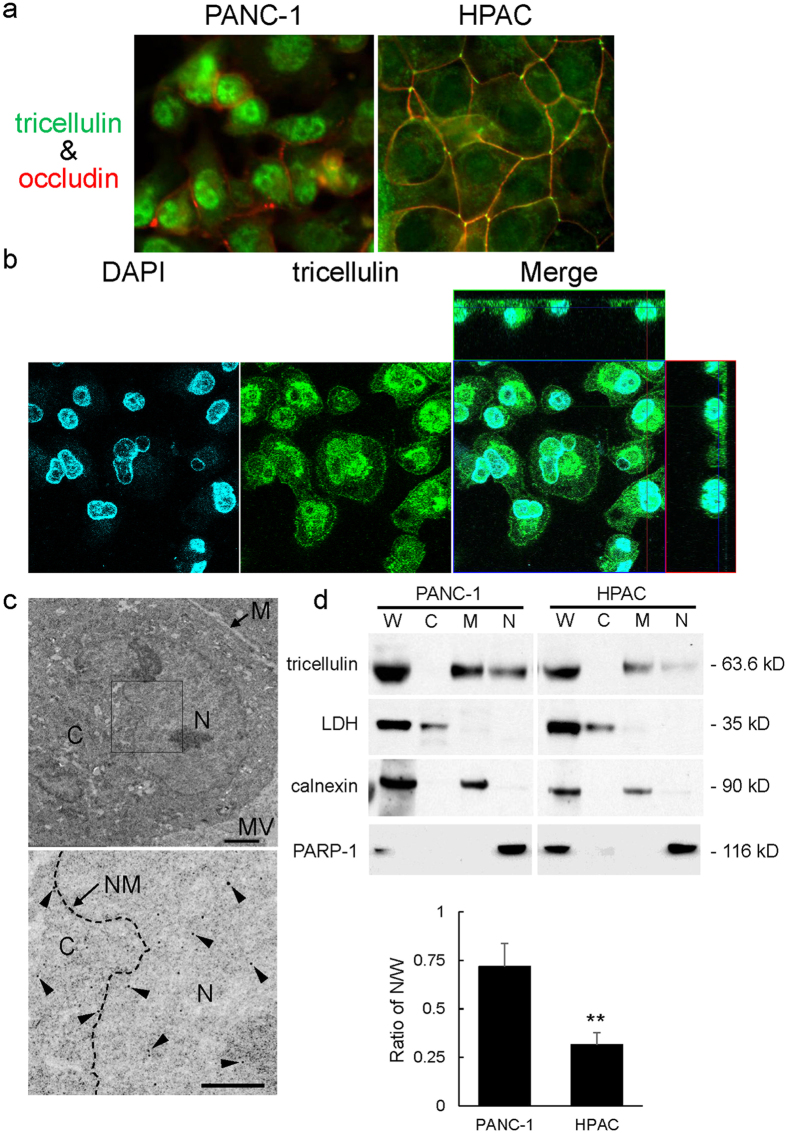
Nuclear localization of tricellulin in poorly differentiated pancreatic cancer cell *in vitro*. (**a**) Immunofluorescence labeling of tricellulin (green) and occludin (red). Differentiated HPAC cells showed tricellulin staining predominantly at the tricellular tight junction, whereas poorly differentiated PANC-1 cells showed tricellulin staining in the nucleus. Occludin was localized at the intercellular boundary in both cell lines. (**b**) Confocal laser microscopic images of tricellulin in PANC-1 cells. Tricellulin co-localized with DAPI in nuclei. (**c**) Immuno-transmission electron microscopic images with anti-tricellulin antibody in PANC-1 cells. The lower panel is an enlarged view of the square area in the upper panel. Presentative immunogold particles (arrowheads) were detected especially at the nucleus and nuclear membrane. C: cytoplasm, N: nucleus, M: membrane, MV: microvilli, NM: nuclear membrane. Bar: 2 μm (upper panel), 1 μm (lower panel). (**d**) Subcellular distributions of tricellulin in PANC-1 and HPAC cells. The ratio of tricellulin in the nuclear fraction and that in the membrane fraction (ratio of N/M) was increased in PANC-1 cells compared with that in HPAC cells. W: whole lysate, C: cytosolic fraction, M: membrane fraction, N: nuclear fraction. Data are represented as means + SD, and statistical significance was assessed by Student’s *t* test. **p < 0.001. Full-length blots are presented in Supplemental Fig. 4.

In HPAC, a cell line derived from moderately differentiated adenocarcinoma, tricellulin immunoreactivity was basically observed along the intercellular boundary just like a TJ marker protein, occludin, and was especially concentrated at a tricellular TJ, as its name means (Fig. 3a). In contrast, in the poorly differentiated cell line PANC-1, tricellulin immunoreactivity was predominantly observed in the nucleus with weak staining at the intercellular boundary (Fig. 3a). In confocal scanning laser microscopic analysis of PANC-1 cells, tricellulin immunoreactivity (Fig. 3b, green) was observed in the nucleus and cytoplasm, including perinuclear areas. It was also seen at the cell borders between adjacent cells. Tricellulin immunoreactivity was co-localized with DAPI (Fig. 3b, blue), a nuclear staining marker. Tricellulin immunoreactivity was observed throughout the cell, including the cytoplasm and cell borders, but was strongly detected in heterochromatin of the nucleus and nuclear membrane by immuno-transmission electron microscopic analysis with the anti-tricellulin antibody (Fig. 3c). Nuclear localization of tricellulin was not obvious in HPAC cell by either of the analyses (data not shown). Subcellular fractionation analysis was performed to compare the subcellular localizations of tricellulin in the two cell lines. In the poorly differentiated cell line PANC-1, tricellulin was detected in the nuclear and membrane fractions but was not detected in the cytosolic fraction (Fig. 3d). On the other hand, in HPAC cells, tricellulin was detected in the membrane fraction and a small amount of tricellulin was observed in the nuclear fraction. Tricellullin in the membrane fraction must correspond to immunoreactivity in the cytoplasm and plasma membrane because the membrane fraction contains the plasma membrane and membranous organelles according to the manufacturer’s instructions. PANC-1 cells showed a pronounced propensity for localization in the nucleus compared to HPAC cells by using the N/M ratio, a signal intensity ratio of tricellulin in the nuclear fraction (N) to that in the membrane fraction (M), which was estimated on the basis of Western blot analysis of biochemical fractionation (Fig. 3d). These results indicated that tricellulin localization shifts from the membrane to the nucleus with decreasing differentiation status *in vitro*.

**Figure 4:**
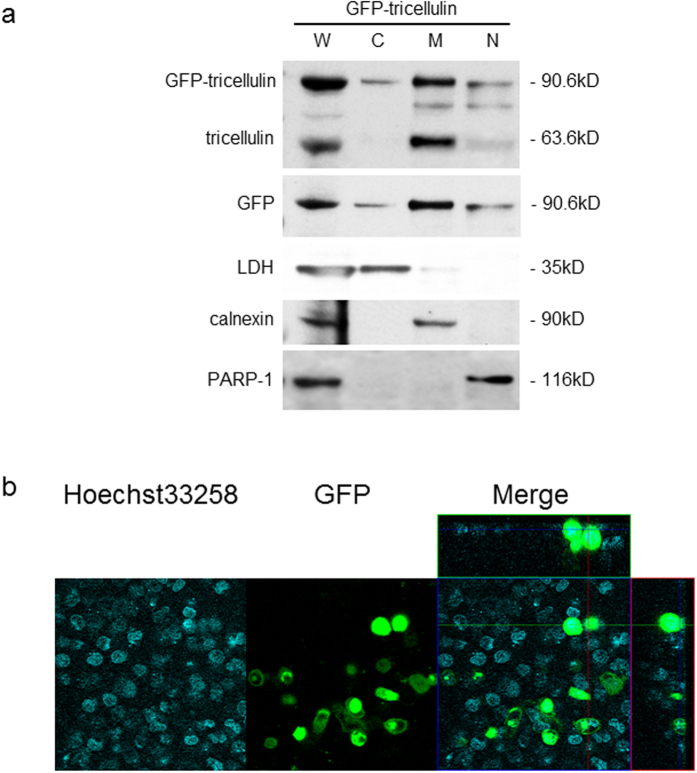
Subcellular distribution of overexpressed GFP-tricellulin in PANC-1 cells. (**a**) Western blot analysis of subcellular fractions from PANC-1 cells overexpressing GFP-tagged tricellulin. GFP-tagged tricellulin was detected in all subcellular fractions including the nuclear fraction. W: whole lysate, C: cytosolic fraction, M: membrane fraction, N: nuclear fraction. Full-length blots are presented in Supplemental Fig. 4. (**b**) Live cell image of PANC-1 cells overexpressing GFP-tagged tricellulin using confocal laser microscopy. GFP-tricellulin co-localized with Hoechst33258 in the nucleus.

In PANC-1 cells, exogenously transfected tricellulin was also localized in the nucleus. We constructed a vector expressing N-terminally GFP-tagged full-length tricellulin (GFP-tricellulin) and transfected it in PANC-1 cells to monitor the localization of exogenously expressed tricellulin (in live cells). Confocal scanning laser microscopic analysis revealed co-localization of GFP-tricellulin and Hoechst33258, a nuclear staining marker, at 48 hours after transfection (Fig. 4a), indicating that tricellulin accumulates in the nucleus of live PANC-1 cells. In Western blot analysis with anti-tricellulin antibody, we confirmed the GFP-tricellulin band (90.6 kDa) in addition to the endogenous tricellulin band (63.6 kDa) in the nuclear fraction from GFP-tricellulin-transfected PANC-1 cells (Fig. 4b). On the other hand, after overexpression of tricellulin in HPAC cells, membrane and cytoplasmic localization of tricellulin was observed by immunofluorescence microscopy, but nuclear localization of tricellulin was not obvious (Supplemental Fig. 2).

### Nuclear localization of tricellulin promotes proliferation and invasiveness of poorly differentiated pancreatic cancer cells *in vitro*

Our preliminary observations suggested that nuclear localization of tricellulin has some roles in tumor progression of poorly differentiated pancreatic cancer cells. To clarify the possible relationship between nuclear localization of tricellulin and malignant behavior in pancreatic neoplasia, tricellulin was overexpressed or its expression was suppressed in PANC-1 cells. Overexpression and suppressed expression of tricellulin were examined by Western blot analysis (Fig. 4a and Supplemental Fig. 3) at 48 hours after transfection of the GFP-tricellulin vector and small interfering RNA (siRNA) for tricellulin, respectively. Nuclear localization of overexpressed GFP-tricellulin was confirmed by confocal scanning laser microscopic analysis as described above (Fig. 4b).

**Figure 5:**
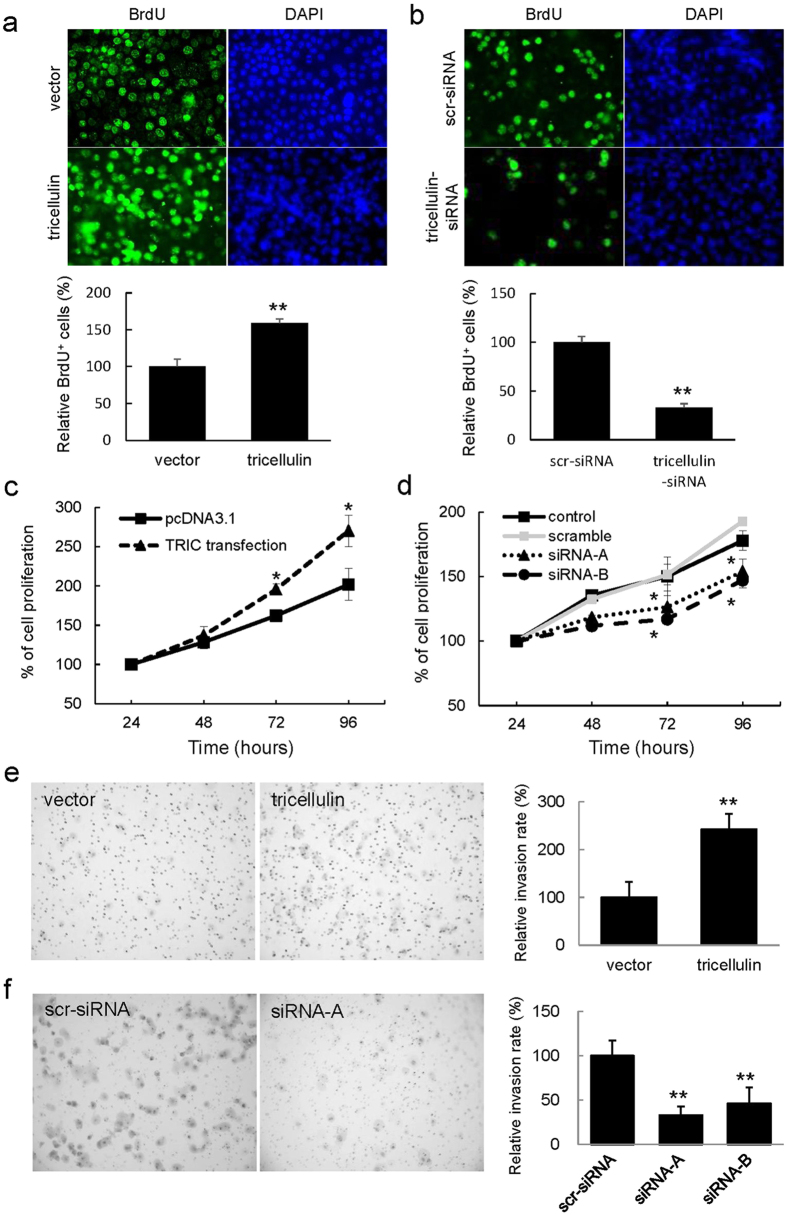
Nuclear localization of tricellulin promotes proliferation and invasiveness of poorly-differentiated pancreatic cancer cell *in vitro.* (**a**,**b**) Representative images of PANC-1 cells in the BrdU incorporation assay (green: BrdU-labeled nuclei). (**c**,**d**) WST-1 assay. (**e**,**f**) Representative images of PANC-1 cells in the Matrigel invasion assay. Tricellulin-overexpressed cells (tricellulin) show enhanced proliferation and invasiveness, whereas cells with suppression of tricellulin expression (tricellulin-siRNA) show reduced proliferation and invasiveness. Scr-siRNA: scrambled siRNA. Data are represented as means ± SD, and statistical significance was assessed by Student’s *t* test. *p < 0.05, **p < 0.001.

The proliferating ability of the cells was evaluated by using the BrdU incorporation assay at 48 hours after transfection (Fig. 5a,b). Tricellulin-overexpressing cells exhibited a significant increase (159%) of BrdU-labeled cells, whereas knockdown of tricellulin significantly reduced the BrdU incorporation ratio to 32.9% of that of control cells. The WST-1 assay also showed that tricellulin overexpression significantly increased cell proliferation, whereas knockdown of tricellulin reduced proliferation of PANC-1 cells (Fig. 5c,d). These results indicated that cell proliferation is positively associated with the amount of nuclear localized tricellulin because overexpressed GFP-tricellulin was clearly observed in the nucleus as described above. In HPAC cells, in which nuclear localization of tricellulin was not obvious, we confirmed that knockdown of tricellulin expression did not affect cell proliferation in a WST-1 assay (data not shown).

To assess the invasive properties, we performed a Matrigel invasion assay (Fig. 5e,f). At 24 hours after plating, there was a significant difference in infiltration efficiency between control and tricellulin knockdown cells, 33% and 46.6%, respectively. In contrast, overexpression of tricellulin dramatically increased the efficiency to 242%, suggesting that nuclear accumulation of tricellulin plays an important role in invasiveness.

**Figure 6:**
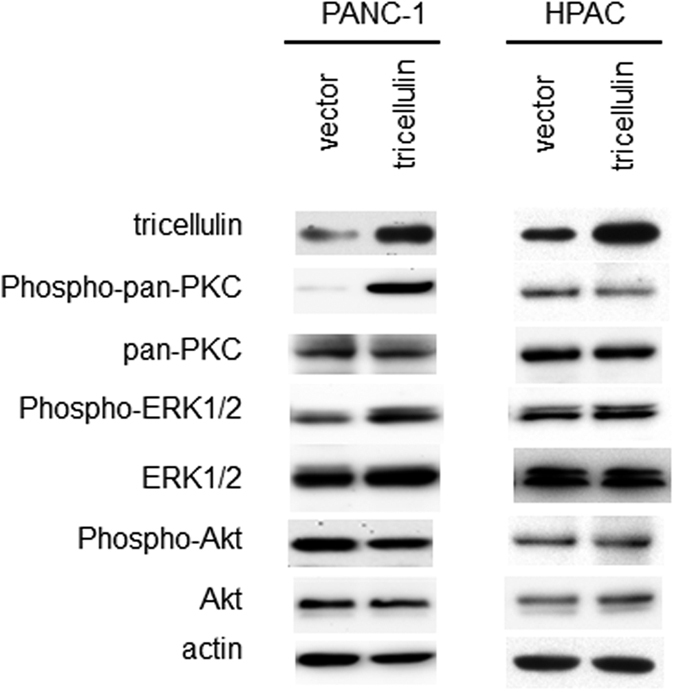
Signaling pathways activated by nuclear localization of tricellulin. Tricellulin overexpression enhanced phosphorylation of PKC and ERK1/2 in PANC-1 cells but not in HPAC cells. Full-length blots are presented in Supplemental Fig. 5.

Finally, we investigated signal transduction pathways that mediate the effects of tricellulin expression on malignant behaviors (Fig. 6). Tricellulin overexpression enhanced phosphorylation of PKC and ERK1/2 in PANC-1 cells but not in HPAC cells. Phospho-Akt level was not affected by tricellulin overexpression in either of the cell lines. These results suggested that tricellulin is preferentially associated with PKC and MAPK signaling pathways that can promote pancreatic neoplasia.

## Discussion

In the present study, we found that nuclear localization of tricellulin is significantly associated with clinicopathological parameters such as lymph node metastasis and shorter survival time. We also demonstrated that nuclear localization of tricellulin promotes malignant behaviors of pancreatic carcinoma cells. Immunohistochemical analyses revealed that immunoreactive intensity of tricellulin was decreased in poorly differentiated adenocarcinomas compared with that in well- and moderately differentiated adenocarcinomas. Furthermore, changes in tricellulin immunostaining were not only its staining intensity but also its localization, from the plasma membrane and cytoplasm (well- and moderately differentiated) to the nucleus (poorly differentiated).

It is plausible that with progression of pancreatic cancer tricellulin alters its expression level and localization as a component of TJ structures in association with destruction of TJs, one of the characteristic phenomena during dedifferentiation. In addition to increased nuclear staining of tricellulin, we also observed that membranous staining was decreased in poorly differentiated adenocarcinoma compared to that in well-differentiated adenocarcinoma, as was reported by Korompay *et al.*^23^. It has been shown that the expression of TJ-associated proteins (TJPs) disperses from the original location in the apical membrane fraction to the cytoplasm in association with progress of epithelial-mesenchymal transition (EMT) or dedifferentiation *in vivo* and *in vitro*^8^. For example, expression of claudin-1 was decreased in pancreatic cancer cell lines compared with that in normal human pancreatic duct epithelial cells, and its expression was observed in the cytoplasm in poorly differentiated pancreatic adenocarcinoma cells^26^. Since the observations indicated that TJPs are useful immunohistochemical markers for the differentiation status of cancer^27^,^28^,^29^,^30^, our results suggest that tricellulin could be included in such markers.

We clearly observed nuclear localization of tricellulin in poorly differentiated adenocarcinomas, though tricellulin has been shown to be a transmembrane protein in the plasma membrane. This is based on published data showing that CLDN-1 mislocalized from the plasma membrane to the nucleus and cytoplasm in metastatic colon cancer cells^31^ and in airway smooth muscle cells^32^. Nuclear distributions of CLDN-2 and E-cadherin were also observed in lung adenocarcinoma cells and pancreatic endocrine tumors, respectively^33^,^34^. The presence of tricellulin in the nucleus was previously reported for HCCs^35^ and hepatoblastoma^36^, but the authors of those reports stated that nuclear localization of tricellulin was randomly observed in only a few cells and they did not analyze the association between nuclear staining and prognosis. In this study, we also observed nuclear localization of tricellulin in pancreatic adenocarcinoma and we also found that the nuclear staining score was associated with differentiation and prognosis. In addition to immunohistochemistry, we confirmed the nuclear localization of tricellulin through a multiple approach including live cell imaging, immunofluorescence, immunoelectron microscopy and biochemical fractionation by using a cell culture model.

Importantly, a high nuclear tricellulin immunoreactive score was correlated with lymph node metastases and lymphatic invasion and reduced overall survival in pancreatic cancer patients. These results may support the explanation that nuclear localized tricellulin contributes to tumor malignancy, including metastatic ability, in pancreatic cancer. This is based on our observations of nuclear localization of tricellulin in a poorly differentiated cancer cell line (PANC-1), a cell line without intact TJ function (unpublished data), but not in a differentiated cancer cell line (HPAC), a cell line with intact TJ function^22^. In addition, overexpression of tricellulin in the nucleus promoted proliferation and invasiveness of PANC-1 cells. The association between expression level of tricellulin and prognosis has been controversial. It has been reported that poor prognosis is linked to high expression of tricellulin in HCC but to low expression of tricellulin in iCCC and hepatoblastoma^35^,^36^. The relationship between expression level of tight junction proteins and prognosis has also been controversial: both increased expression and decreased expression have been reported to be associated with poor prognosis in various cancers^37^,^38^,^39^. Each of the tight junction molecules, including tricellulin, may play different roles depending on tissue type or tumor grade. For a better understanding of each molecule, its subcellular localization should be evaluated in addition to immunoreactive intensity.

Since it is unlikely to translocate to the nucleus, the question of what mechanism regulates the translocation of tricellulin to the nucleus has remained to be answered. Although underlying mechanisms have not been fully established, several transmembrane proteins of TJs and adherens junctions were observed to be mislocalized to the nucleus in some cancer cells. Unexpectedly, we did not detect any classical nuclear localization signal (NLS) sequences in the human tricellulin sequence by searching with nuclear localization software-based analysis (Nuclear Localization Signal database, http://rostlab.org/services/nlsdb/). We then hypothesized that tricellulin may interact with an unknown partner molecule(s) with an NLS sequence to go to the nucleus or translocate to the nucleus via some importin-independent system. In the case of claudin-2, its nuclear distribution is up-regulated by dephosphorylation and serves to retain ZONAB and cyclin D1 in the nucleus, resulting in the enhancement of proliferation of lung adenocarcinoma cells^33^. We did not observe interaction between tricellulin and ZO-1, a TJP with NLS sequences, by immunoprecipitation analyses (data not shown). Our studies, however, have not fully proven the significance of the nuclear localization of tricellulin in tumor progression, whether it is a causative event or just a bystander. Further studies are needed to clarify the molecular mechanism underlying the nuclear localization of tricellulin in poorly differentiated pancreatic cancer and how its nuclear localization contributes to pancreatic neoplasia via such signaling pathways. The combination of immunoprecipitation and mass spectrometry may reveal a molecule(s) that interacts with tricellulin in the nucleus.

Another question is how nuclear localized tricellulin contributes to tumor malignancy, because there has been no report about tricellulin function in the nucleus. In this study, we observed that nuclear overexpression of tricellulin induced activation of MAPK and PKC pathways, but not the Akt pathway, in a poorly differentiated pancreatic cancer cell line. Activation of MAPK and PKC pathways is generally supported in malignant pancreatic tumors, and a large number of molecules are involved in activation of the pathways^1^,^2^,^3^. Our results suggested that nuclear localized tricellulin promotes cell proliferation and invasiveness possibly in association with MAPK1 and PKC pathways in pancreatic cancers, although detailed mechanisms remain to be elucidated.

In conclusion, poorly differentiated pancreatic carcinomas show mislocalization of tricellulin from the plasma membrane to nuclei, which is significantly correlated with poor survival and tumor malignancies. Our results provide new insights into the function of the TJP tricellulin, and its nuclear localization may become a new prognostic factor for pancreatic cancers.

## Materials and Methods

### Antibodies

Primary antibodies used in this study include the following: rabbit polyclonal anti-tricellulin (c-term), mouse monoclonal anti-occludin (Zymed Laboratories, San Francisco, CA); rabbit polyclonal anti-actin (Sigma-Aldrich, St Louis, MO); goat polyclonal anti-LDH (as a marker enzyme of cytosolic fraction), mouse monoclonal anti-calnexin (as a marker of the membrane fraction) (Abcam, Cambridge, MA); mouse monoclonal anti-PAPR-1 (as a marker of the nuclear fraction) (Calbiochem). Twelve-nm colloidal gold-conjugated anti-rabbit IgG was purchased from Jackson Immuno Research Laboratories (Western Grove, PA). The secondary antibodies used were horseradish peroxidase (HRP)-conjugated anti-rabbit or anti-mouse immunoglobulin (Ig)G (Dako ChemMate, Glostrup, Denmark), Alexia Flour 488 (green)-labeled anti-rabbit or anti-mouse IgG (Invitrogen, Carlsbad, CA), and Alexa Flour 594 (red)-labeled anti-rabbit or anti-mouse IgG (Invitrogen). DAPI was obtained from Sigma-Aldrich and Hoechst 33342 solution was obtained from Dojindo (Kumamoto, Japan).

### Immunohistochemistry for primary pancreatic adenocarcinoma tissues

A total of 65 cases of operated pancreatic adenocarcinoma were obtained from Sapporo Medical University Hospital, Japan. Written informed consent was obtained from all patients. The study was approved by the ethics committee of Sapporo Medical University. All methods were performed in accordance with the relevant guidelines and regulations of the University. Hematoxylin and eosin-stained slides from each case were reviewed. We determined glandular differentiation of the tumor according to the guidelines of the WHO classification^40^. According to the evaluation by three pathologists, 17, 28 and 20 cases were verified as well-, moderately and poorly differentiated adenocarcinoma, respectively.

Immunostaining of tricellulin was performed as described previously^14^ using the primary polyclonal anti-tricellulin antibody. Antigen retrieval was performed by immersing sections in 10 mM Tris - 1 mM EDTA buffer (pH 9.0) and boiling them in a microwave (95 °C, 30 min). Immunostaining was performed using the Dako REAL^TM^ EnVision^TM^ Detection System (Dako ChemMate, Glostrup, Denmark) and diaminobenzidine (Dako Laboratories, Carpentaria, CA, USA) as a chromogen according to the manufacturer’s instructions. Sections were then counterstained with hematoxylin, dehydrated, and mounted.

Immunoreactivity was assessed by three pathologists. In cases of disagreement, the case was re-evaluated and a consensus agreement on the reactivity was made. The score of membranous or cytoplasmic immunoreactivity was determined from the total of the tricellulin-positive area and the intensity of immunoreactivity. The tricellulin-positive area was assessed semiquantitatively as follows: 0–25% (0), 26–50% (1), 51–75% (2), and 76–100% (3). The intensity of immunoreactivity was assessed semiquantitatively as follows: weak (1), moderate (2), and strong (3). For scoring nuclear immunoreactivity, we counted the tricellulin-positive nuclei in 10 randomly selected microscopic fields. Data were analyzed using the ROC test, Student’s t-test, ANOVA test and Pearson’s correlation coefficient. Data analysis was performed using SPSS software version 20 (SPSS, Chicago, Illinois, USA).

### Cell lines and cell culture

Human pancreatic cancer cell lines PANC-1 and HPAC were purchased from ATCC (Manassas, VA) and maintained in DMEM (Sigma-Aldrich, St Louis, MO) supplemented with 10% fetal bovine serum (FBS, Invitrogen, Carlsbad, CA). The medium for the cell lines contained 100 U/ml penicillin and 100 μg/ml streptomycin, and all cells were plated on 35- or 60-mm culture dishes (Corning Glass Works, Corning, NY) that were coated with rat tail collagen (500 μg of dried tendon/ml in 0.9% acetic acid) and incubated in a humidified 5% CO_2_ incubator at 37 °C.

### Transfection and RNAi

Fifteen μg of pcDNA3.1(+)-GFP-tricellulin, a vector expressing N-terminally GFP-tagged full-length human tricellulin, was transfected into PANC-1 cells using Lipofectamine 2000 (Invitrogen, Carlsbad, CA, USA). After 6 h of incubation, the cells were transferred to DMEM medium containing 10% FBS. All cell cultures were maintained for 48 h after the transfection and then examined by the following assays. The cells were stained with 1 μM Hoechst 33342 (Dojindo, Kumamoto, Japan) and observed under a confocal laser scanning microscope (ConfoCor3LSM510META; Carl Zeiss, Jena, Germany).

For RNA interference studies, small interference RNAs (siRNAs) duplex targeting the mRNA sequences of human tricellulin and scrambled Stealth RNAi were purchased from Invitrogen (Carlsbad, CA). One day before transfection, PANC-1 cells were plated in a medium without antibiotics so that they would be half confluent at the time of transfection. The cells were transfected with 100 nM siRNAs using Lipofectamine RNAiMAX (Invitrogen) as a carrier according to the manufacturer’s instructions.

### Immunofluorescence microscopy

For immunocytochemistry, cells were grown on 35-mm glass-base dishes (Iwaki, Chiba, Japan) coated with rat tail collagen. They were fixed with an ethanol and acetone 1:1 mixture at −20 °C for 10 min. Staining was performed as described previously^22^ using the primary polyclonal anti-tricellulin antibody and monoclonal anti-occludin antibody. The specimens were examined using an epifluorescence microscope (Olympus, Tokyo, Japan) and a confocal laser scanning microscope (LSM510; Carl Zeiss, Jena, Germany).

### Subcellular fractionation and Western blot analysis

By using a ProteoExtract^TM^ Subcellular Proteome Extraction Kit (Calbiochem, Darmstadt, Germany), subcellular fractions of cytosols, membranes and nuclei were collected according to the manufacturer’s instructions. Western blot analysis was performed as described previously^22^. To verify the purity of each fraction, the following antibodies were used as markers in the Western blot analysis: anti-LDH as a marker enzyme of the cytosolic fraction, anti-calnexin as a marker of the membrane fraction, and anti-PAPR-1 as a marker of the nuclear fraction.

### Immuno-transmission electron microscopy (TEM) analysis

Immuno-transmission electron microscopy (TEM) was performed as described previously^22^ using the primary polyclonal anti-tricellulin antibody. PANC-1 cells were cultured on 60-mm dishes. The cells were scraped from the dishes and collected in microcentrifuge tubes. After fixation by 4% paraformaldehyde, the samples were immediately frozen in liquid nitrogen. Mounted samples were cut into 20-μm-thick sections with a cryostat. Non-specific reactions were blocked with 10% bovine serum albumin (Sigma Co., Tokyo, Japan), and sections were then incubated with the anti-tricellulin (1:100) antibody overnight at 4 °C. After incubation, the specimens were incubated with a biotinylated secondary antibody (LSAB2 Kit, Dako A/S, Copenhagen, Denmark). The specimens were fixed with 1.0% glutaraldehyde for 15 min at 4 °C and incubated with 0.05 M HEPES solution. After incubation, they were enhanced with a silver enhancement kit (Jackson Immuno Research Laboratories). The specimens were fixed with 1.0% OsO4 in PBS at 4 °C for 1 h, dehydrated through a graded ethanol series, embedded in epoxy resin, and cut into ultrathin sections with a Sorvall MT5000 ultramicrotome (DEDuPontUPOMT, New Castle, DE, USA). The ultrathin sections were stained with uranyl acetate and lead citrate and examined with a transmission electron microscope (JEM-1400; JEOL Ltd., Tokyo, Japan).

### Cell proliferation and invasion assays

Incorporation of 5-bromo-2-deoxyuridine (BrdU) (Sigma-Aldrich, St Louis, MO) into cell DNA was used to determine proliferation rate. Cells were grown on 35-mm glass-base dishes (Iwaki, Chiba, Japan) coated with rat tail collagen. The cells were incubated for 2 h after treatment with 20 μM BrdU and were then fixed in cold absolute ethanol. After the samples had been incubated with 2 N HCl at room temperature for 20 min, they were incubated with monoclonal anti-BrdU antibody (Dako, Santa Barbara, CA, USA) (1:100) at room temperature for 1 h and then with Alexa 488 (green)-conjugated anti-mouse IgG antibody (1:100) at room temperature for 1 h. DAPI (Sigma-Aldrich, St Louis, MO) was used for counterstaining of nuclei in the cells. The number of cells with BrdU-labeled nuclei was counted using an epifluorescence microscope (Olympus, Tokyo, Japan). More than 1000 cells were counted per dish, and three dishes were examined per experiment.

PANC-1 cells were seeded in 96-well plates. After 24 h of incubation, cell viability was assessed by using a cell counting kit-8 (Dojindo) according to the manufacturer’s instructions. Absorbance at a wavelength of 450 nm was measured using a Bio-Rad Model 680 microplate reader (Bio-Rad).

For the invasion assay, we used a Corning BioCoat Matrigel Invasion Chamber (Fisher Scientific) according to the manufacturer’s instructions. PANC-1 cells in a medium without FBS were plated onto the Matrigel chambers at 48 h after transfection. The lower chamber of the Transwell was filled with medium containing 10% FBS. At 24 h after plating, invading cells were fixed and visualized by 0.04% Crystal violet and 2% ethanol for 10 min. The areas of invading cells were measured using a microscope imaging system (Olympus).

### Statistical analysis

In immunohistochemical analysis of human tissues, the measured values are presented as medians and inter-quartile ranges (25–75%). Correlations between number of tricellulin-positive nuclei and clinicopathological variables were assessed by the Mann-Whitney test, Kruskal-Wallis test or Kruskal-Wallis test with post hoc Steel-Dwass test. Survival rates were calculated by the Kaplan-Meier method and compared by the log-rank test. In all other analyses, the measured values are presented as means ± SD. Statistical significance of differences was evaluated using the unpaired Student’s t-test.

All statistical calculations were performed with the use of SPSS statistics ver. 20.

## Additional Information

**How to cite this article**: Takasawa, A. *et al.* Nuclear localization of tricellulin promotes the oncogenic property of pancreatic cancer. *Sci. Rep.* **6**, 33582; doi: 10.1038/srep33582 (2016).

## Supplementary Material

Supplementary Information

## Figures and Tables

**Table 1:** Immunoreactivity of tricellulin in pancreatic adenocarcinoma tissues.

		Localization			
		Membrane	Cytoplasm	Nuclei	
**a: Comparison of the immunoreactive score and localization according to tumor differentiation status.**					
Differentiation	Well	1.0 (0.0–2.0)	5.0 (2.0–9.0)	7.0 (2.0–16.0)	
	Moderate	0.0 (0.0–3.3)	6.0 (3.0–9.0)	11.0 (4.8–26.6)	
	Poor	0.0 (0.0–0.0)	4.8 (2.8–6.4)	118.0 (21.0–247.0)*	
**b: Comparison of the number of tricellulin-positive nuclei according to tumor differentiations status**					
		**Differentiation**			**Total**
		**Well**	**Moderate**	**Poor**	
Number of positive nuclei	< = 20.00	13	22	4	40
	20.00<	4*	6*	16*	25
Total		17	28	20	65

Data are represented as medians and inter-quartile ranges (25–75%), and statistical significance was assessed by the Kruskal-Wallis test with post hoc Steel-Dwass test *p < 0.001 (vs Well), p = 0.002 (vs Moderate). Statistical significance was assessed by the chi-square test. *p < 0.001.

**Table 2:** Nuclear distribution of tricellulin and clinicopathological features of pancreatic adenocarcinomas.

Factors		Total	Median	Inter-quartile range	P value
Sex	FM	2936	1324	4–32M9–200	0.0929–200(Mann-Whitney test)
Age(median)	< = 6970+	3332	1521	4.5–1458–74	0.909(Mann-Whitney test)
Size(median)	< = 30310+	3530	12.523	4–48.58.25–123.5	0.161(Mann-Whitney test)
pTcategory	123	5228	211121	0–8.522–2006.5–104	0.124 (1 vs 2)0.023 (1 vs 3)0628 (2 vs 3)(Kruskal-Wallis test with post hoc Steel-Dwass test)
pNcategory	01	1748	523	2.5–18.759.75–134.5	0.004(Mann-Whitney test)
UICCstage	1234	221348	8102.51521	7–95–2002–4.956–118	0.279(Kruskal-Wallis test)
Lymphaticinvasion	negativepositive	1352	1421	2.75–23.56–118	0.058(Mann-Whitney test)
Vesselinvasion	negativepositive	1649	1023	4–19.756.5–134.5	0.057(Mann-Whitney test)
Neuralinvasion	negativepositive	461	14415.5	21–286.55–69.75	0.071(Mann-Whitney test)
Tumorlocation	PbPbtPhPh(U)PhbPhbtPt	83383517	11318.523237	4–155.52.5–13.56.75–11818–161.51–216.5–81.5	0.143(Kruskal-Wallis test)

## References

[b1] Hezel A. F., Kimmelman A. C., Stanger B. Z., Bardeesy N. & DePinho R. A. Genetics and biology of pancreatic ductal adenocarcinoma. Genes Dev. 20, 1218–1249 (2006). 16702400 10.1101/gad.1415606

[b2] Siegel R. *et al.* Cancer statistics, 2011: the impact of eliminating socioeconomic and racial disparities on premature cancer deaths. CA Cancer J. Clin. 61, 212–236 (2011). 21685461 10.3322/caac.20121

[b3] Hidalgo M. Pancreatic cancer. N. Engl. J. Med. 362, 1605–1617 (2010). 20427809 10.1056/NEJMra0901557

[b4] Schneeberger E. E. & Lynch R. D. Structure, function, and regulation of cellular tight junctions. Am. J. Physiol. 262(6) (Pt 1), L647–661 (1992). 1616050 10.1152/ajplung.1992.262.6.L647

[b5] Gumbiner B. M. Breaking through the tight junction barrier. J. Cell Biol. 123(6) (Pt 2), 1631–1633 (1993). 8276885 10.1083/jcb.123.6.1631 PMC2290864

[b6] Cereijido M., Valdes J., Shoshani L. & Contreras R. G. Role of tight junctions in establishing and maintaining cell polarity. Annu. Rev. Physiol. 60, 161–177 (1998). 9558459 10.1146/annurev.physiol.60.1.161

[b7] Tsukita S., Furuse M. & Itoh M. Multifunctional strands in tight junctions. Nat. Rev. Mol. Cell Biol. 2(4), 285–293 (2001). 11283726 10.1038/35067088

[b8] Sawada N. *et al.* Tight junctions and human diseases. Med. Electron Microsc. 36(3), 147–156 (2003). 14505058 10.1007/s00795-003-0219-y

[b9] Schneeberger E. E. & Lynch R. D. The tight junction: a multifunctional complex. Am. J. Physiol. Cell Physiol. 286(6), C1213–C1228 (2004). 15151915 10.1152/ajpcell.00558.2003

[b10] Akimoto T. *et al.* Analysis of the expression and localization of tight junction transmembrane proteins, claudin-1, -4, -7, occludin and JAM-A, in human cervical adenocarcinoma. Histol. Histopathol. 31(8), 921–931 (2016). 26847087 10.14670/HH-11-729

[b11] Maeda T. *et al.* Claudin-4-targeted therapy using Clostridium perfringens enterotoxin for prostate cancer. Prostate 72(4), 351–360 (2012). 21656836 10.1002/pros.21436

[b12] Tokes A. M. *et al.* Claudin-1, -3 and -4 proteins and mRNA expression in benign and malignant breast lesions: a research study. Breast Cancer Res. 7(2), R296–305 (2005). 15743508 10.1186/bcr983 PMC1064136

[b13] Cheung S. T. *et al.* Claudin-10 expression level is associated with recurrence of primary hepatocellular carcinoma. Clin. Cancer Res. 11(2) (Pt 1), 551–556 (2005). 15701840

[b14] Keira Y. *et al.* An immunohistochemical marker panel including claudin-18, maspin, and p53 improves diagnostic accuracy of bile duct neoplasms in surgical and presurgical biopsy specimens. Virchows Arch. 466(3), 265–277 (2015). 25503275 10.1007/s00428-014-1705-4

[b15] Lodi C. *et al.* Claudin-4 differentiates biliary tract cancers from hepatocellular carcinomas. Mod. Pathol. 19(3), 460–469 (2006). 16439986 10.1038/modpathol.3800549

[b16] Ding L., Lu Z., Lu Q. & Chen Y. H. The claudin family of proteins in human malignancy: a clinical perspective. Cancer Manag. Res. 5, 367–375 (2013). 24232410 10.2147/CMAR.S38294 PMC3825674

[b17] Karanjawala Z. E. *et al.* New markers of pancreatic cancer identified through differential gene expression analyses: Claudin 18 and Annexin A8. Am. J. Surg. Pathol. 32(2), 188–196 (2008). 18223320 10.1097/PAS.0b013e31815701f3 PMC2678811

[b18] Soini Y. *et al.* Expression of claudins 7 and 18 in pancreatic ductal adenocarcinoma: association with features of differentiation. J. Clin. Pathol. 65(5), 431–436 (2012). 22396552 10.1136/jclinpath-2011-200400

[b19] Pauli B. U., Weinstein R. S., Alroy J. & Arai M. Ultrastructure of cell junctions in FANFT-induced urothelial tumors in urinary bladder of Fischer rats. Lab. Invest. 37, 609–621 (1977). 599905

[b20] Zhong Y. *et al.* Sequential decrease in tight junctions as revealed by 7H6 tight junction-associated protein during rat hepatocarcinogenesis. Jpn. J. Cancer Res. 85, 351–356 (1994). 8200847 10.1111/j.1349-7006.1994.tb02366.x PMC5919476

[b21] Ikenouchi J. *et al.* Tricellulin constitutes a novel barrier at tricellular contacts of epithelial cells. J. Cell Biol. 171(6), 939–945 (2005). 16365161 10.1083/jcb.200510043 PMC2171318

[b22] Takasawa A. *et al.* Behavior of tricellulin during destruction and formation of tight junctions under various extracellular calcium conditions. Cell Tissue Res. 351(1), 73–84 (2013). 23073616 10.1007/s00441-012-1512-7 PMC3536962

[b23] Korompay A. *et al.* Tricellulin expression in normal and neoplastic human pancreas. Histopathology 60(6B), E76–86 (2012). 22394074 10.1111/j.1365-2559.2012.04189.x

[b24] Yamaguchi H. *et al.* Transcriptional control of tight junction proteins via a protein kinase C signal pathway in human telomerase reverse transcriptase-transfected human pancreatic duct epithelial cells. Am. J. Pathol. 177(2), 698–712 (2010). 20566751 10.2353/ajpath.2010.091226 PMC2913360

[b25] Kojima T. *et al.* c-Jun N-terminal kinase is largely involved in the regulation of tricellular tight junctions via tricellulin in human pancreatic duct epithelial cells. J. Cell Physiol. 225(3), 720–733 (2010). 20533305 10.1002/jcp.22273

[b26] Kyuno D. *et al.* Protein kinase Cα inhibitor protects against downregulation of claudin-1 during epithelial-mesenchymal transition of pancreatic cancer. Carcinogenesis 34, 1232–1243 (2013). 23389293 10.1093/carcin/bgt057

[b27] Shin K., Fogg V. C. & Margolis B. Tight junctions and cell polarity. Annu. Rev. Cell Dev. Biol. 22, 207–235 (2006). 16771626 10.1146/annurev.cellbio.22.010305.104219

[b28] Matter K., Aijaz S., Tsapara A. & Balda M. S. Mammalian tight junctions in the regulation of epithelial differentiation and proliferation. Curr. Opin. Cell Biol. 17, 453–458 (2005). 16098725 10.1016/j.ceb.2005.08.003

[b29] Suh Y. *et al.* Claudin-1 induces epithelial–mesenchymal transition through activation of the c-Abl-ERK signaling pathway in human liver cells. Oncogene 32, 4873–4882 (2013). 23160379 10.1038/onc.2012.505

[b30] Philip R. *et al.* Caludin-7 promotes the epithelial – mesenchymal transition in human colorectal cancer. Oncotarget 6(4), 2046–2063 (2014). 25514462 10.18632/oncotarget.2858 PMC4385835

[b31] Dhawan P. *et al.* Claudin-1 regulates cellular transformation and metastatic behavior in colon cancer. J. Clin. Invest. 115, 1765–1776 (2005). 15965503 10.1172/JCI24543 PMC1150288

[b32] Fujita H. *et al.* Claudin-1 expression in airway smooth muscle exacerbates airway remodeling in asthmatic subjects. J. Allergy Clin. Immunol. 127(6), 1612–1621.e8 (2011). 21624620 10.1016/j.jaci.2011.03.039

[b33] Ikari A. *et al.* Nuclear distribution of claudin-2 increases cell proliferation in human lung adenocarcinoma cells. Biochim. Biophys. Acta 1843, 2079–2088 (2014). 24907662 10.1016/j.bbamcr.2014.05.017

[b34] Chetty R., Serra S. & Asa S. L. Loss of membrane localization and aberrant nuclear E-cadherin expression correlates with invasion in pancreatic endocrine tumors. Am. J. Surg. Pathol. 32(3), 413–419 (2008). 18300809 10.1097/PAS.0b013e31813547f8

[b35] Somorácz A. *et al.* Tricellulin expression and its prognostic significance in primary liver carcinomas. Pathol. Oncol. Res. 20(4), 755–764 (2014). 24652413 10.1007/s12253-014-9758-x

[b36] Schlachter K. *et al.* High tricellulin expression is associated with better survival in human hepatoblastoma. Histopathology. 65(5), 631–641 (2014). 24735023 10.1111/his.12436

[b37] Gonzalez-Mariscal L. *et al.* Role of tight junctions in cell proliferation and cancer. Prog. Histochem. Cytochem. 42, 1–57 (2007). 17502225 10.1016/j.proghi.2007.01.001

[b38] Lal-Nag M. & Morin P. J. The claudins. Genome Biol. 10, 235 (2009). 19706201 10.1186/gb-2009-10-8-235 PMC2745760

[b39] Ding L. *et al.* The claudin family of proteins in human malignancy: a clinical perspective. Cancer Manag. Res. 5, 367–375 (2013). 24232410 10.2147/CMAR.S38294 PMC3825674

[b40] Hruban R. H. *et al.* Ductal adenocarcinoma of the pancreas. In: Bosman F. T., Carneiro F., Hruban R. H., Theise N. D. eds WHO Classification of Tumours of the Digestive System, 4th ed. Lyon, France: International Agency for Research on Cancer. 281–291 (2010).

