# Supplementary material for: Nuclear localization of tricellulin promotes the oncogenic property of pancreatic cancer
Source: Sci Rep. 2016 Sep 19;6:33582. doi: 10.1038/srep33582 (PMC5027560; doi:10.1038/srep33582)
Supplement: Supplementary Information [file srep33582-s1.pdf]

Nuclear localization of tricellulin promotes the oncogenic property of pancreatic cancer

Akira Takasawa[1], Masaki Murata[1], Kumi Takasawa[1], Yusuke Ono[1], Makoto Osanai[1], Satoshi Tanaka[1], Masanori Nojima[2], Tsuyoshi Kono[3], Koichi Hirata[3], Takashi Kojima[4], Norimasa Sawada[1]

1) Departments of Pathology, Sapporo Medical University School of Medicine, Sapporo 608556, Japan
2) Division of Advanced Medicine Promotion, The Advanced Clinical Research Center, The Institute of Medical Science, The University of Tokyo, Tokyo 108-8639, Japan
3) Departments of Surgery, Sapporo Medical University School of Medicine, Sapporo 608556, Japan
4) Department of Cell Science, Research Institute of Frontier Medicine, Sapporo Medical University School of Medicine, Sapporo 608556, Japan

Corresponding author: Masaki Murata
Nishi 16-chome, Minami 1-jo, Chuo-ku, Sapporo-shi, Hokkaido 060-8556, JAPAN
Tel: +81-11-611-2111 (Ext 2701)
FAX: +81-011-613-5665
E-mail: mmurata@sapmed.ac.jp

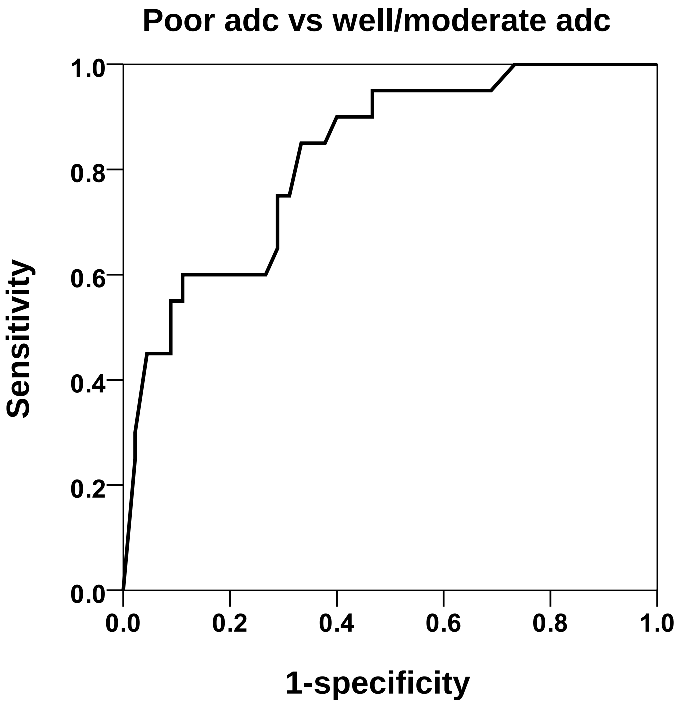

Supplemental Fig. 1. ROC curve analysis of the nuclear tricellulin immunoreactive score in surgical specimens of pancreatic adenocarcinomas. Cutoff value was calculated from the ROC curve to distinguish poorly differentiated adenocarcinoma from well-differentiated and moderately differentiated adenocarcinoma.

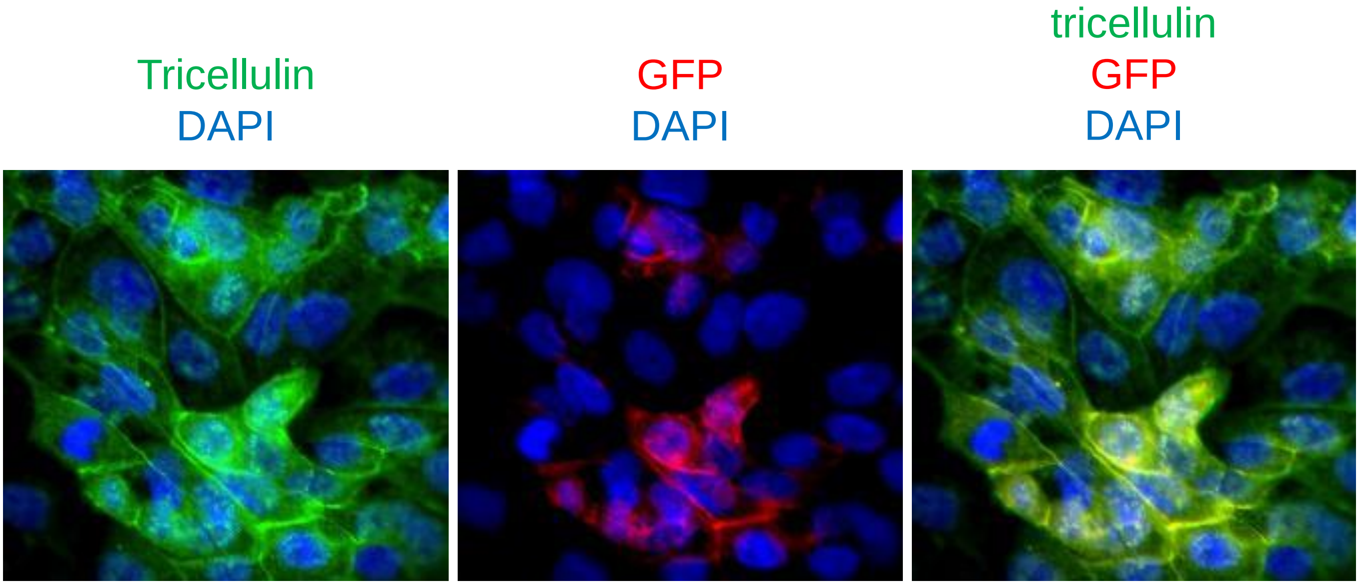

Supplemental Fig. 2. Immunofluorescence labeling of tricellulin (green) and GFP (red) at 72 hours after transfection of the GFP-tricellulin vector in HPAC cells. Membrane and cytoplasmic localization of overexpressed GFP-tricellulin was confirmed by immunofluorescence microscopy.

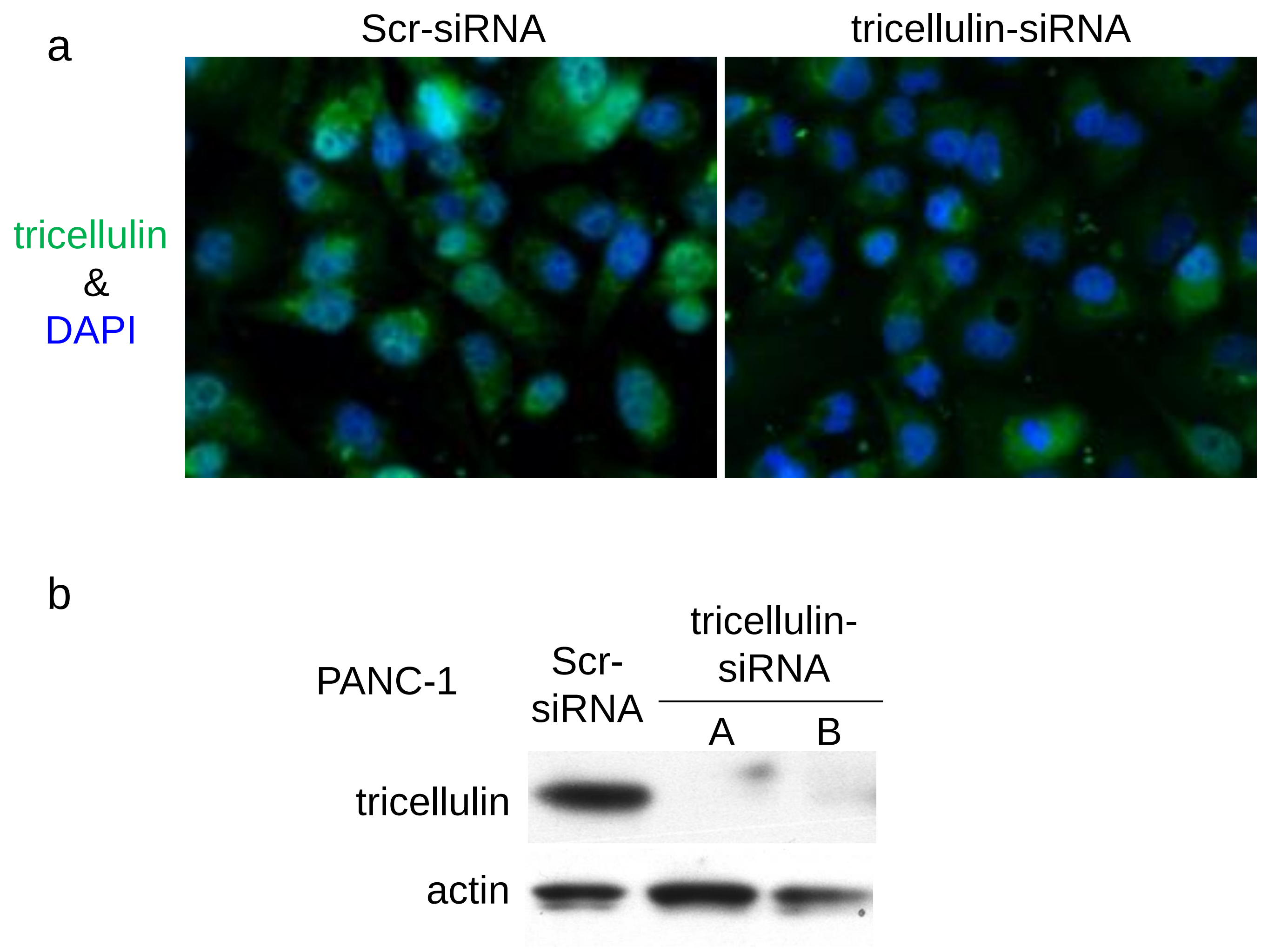

Supplemental Fig. 3. Tricellulin expression in PANC-1 cells after tricellulin knockdown by siRNAs. Immunostaining (a) and Western blotting (b).

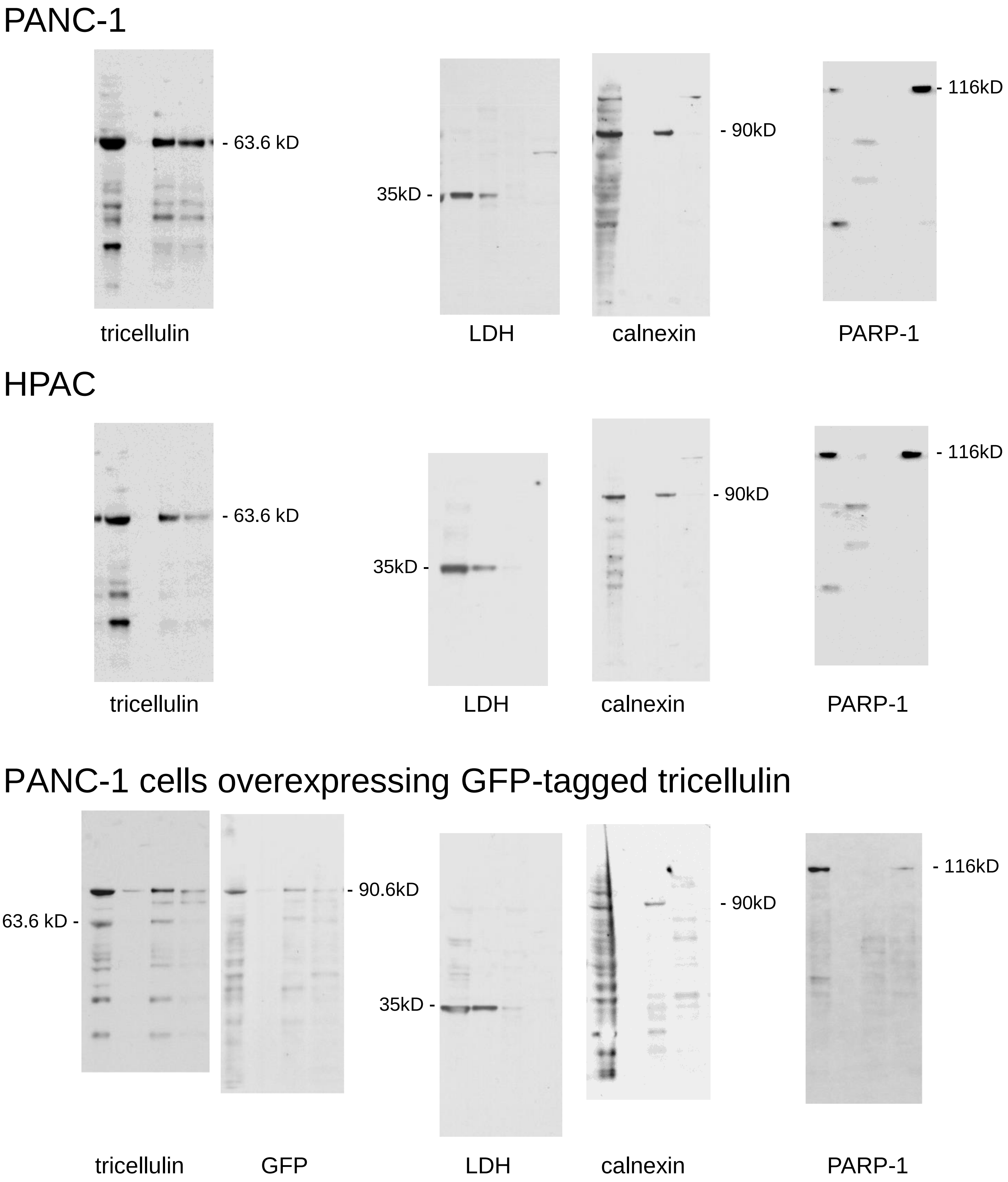

Supplemental Fig. 4. Full-length blots of Figure 3 and 4.

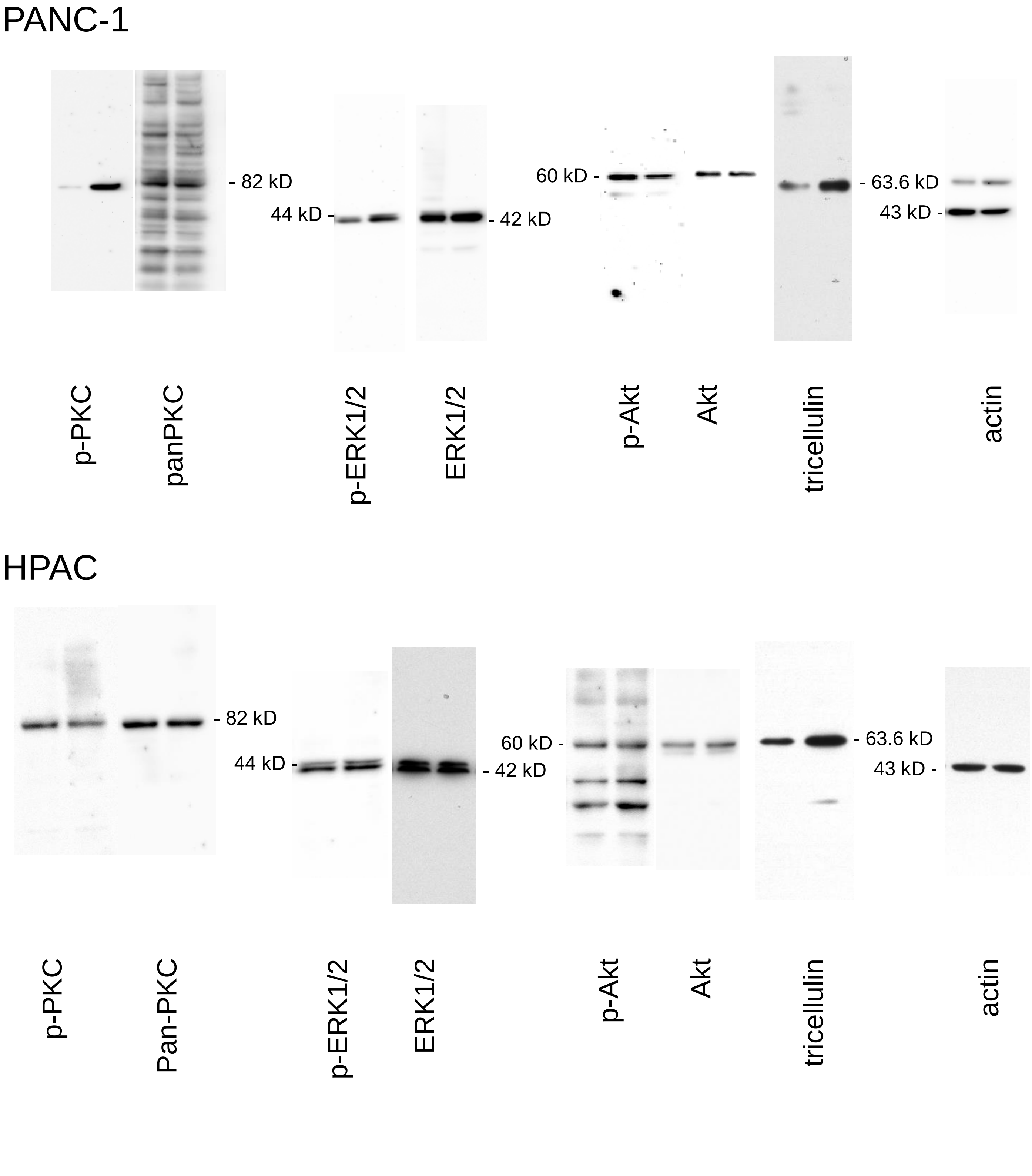

Supplemental Fig. 5. Full-length blots of Figure 5.
